# Assessing the Practical Constraints and Capabilities of Accelerator-Based Focused Ion Thermal Analysis

**DOI:** 10.3390/ma18071514

**Published:** 2025-03-27

**Authors:** Rijul R. Chauhan, Artur Santos Paixao, Benjamin E. Mejia Diaz, Frank A. Garner, Lin Shao

**Affiliations:** Department of Nuclear Engineering, Texas A&M University, College Station, TX 77845, USA; chauriju@tamu.edu (R.R.C.); paixao@tamu.edu (A.S.P.); bmejiad@tamu.edu (B.E.M.D.); fgarnerwnc@tamu.edu (F.A.G.)

**Keywords:** proton irradiation, IR, thermal property, finite element analysis

## Abstract

This study investigates the capabilities of accelerator-based Focused Ion Thermal Analysis (FITA), a remote nondestructive method developed for characterizing thermal properties using a proton beam as a localized heat source. Employing infrared (IR) imaging, FITA captures the evolution of temperature in material samples after the beam is deactivated, enabling precise extraction of thermal properties. However, the performance of FITA is inherently influenced by the IR camera’s resolution and frame rate, which imposes constraints on the types of materials that can be effectively analyzed. Here, a comprehensive series of finite element analysis (FEA) simulations were performed to evaluate the applicability of FITA for a wide range of materials. These simulations assess how variations in IR camera specifications impact the effectiveness of FITA in analyzing different materials. Our findings show that the current method can characterize a wide range of materials, including the majority of nuclear materials typically used in the nuclear industry.

## 1. Introduction

The thermal properties of nuclear materials and their potential degradation under irradiation are critical factors in nuclear reactor applications. Moreover, the fidelity of thermal models and simulations, which concern reactor designs and safety, depends extensively on accurate thermal data. Nuclear materials encompass a wide range, including ceramics such as uranium oxide used as fuel and metals like Zircaloy employed as fuel cladding. Traditionally, the measurement of thermal properties has relied heavily on heat transfer methods—such as the laser flash method [1], various thermoelectric-based techniques [2], the transient plane source method [3], and the hot-wire method [4]. The laser flash method is one of the most popular methods, where laser-induced heat transfer through a specimen of precisely known thickness is measured by tracking temperature readings on the specimen backside. This method demands good thickness, uniformity, and a specific thickness range. For nuclear material testing, this approach often involves measuring individual samples that have undergone radiation damage at specified doses.

Our research group previously reported on the development of a novel remote nondestructive method known as Focused Ion Thermal Analysis (FITA) [5,6]. The first reported demonstration of this technique proceeded on a z-cut single crystal quartz substrate using an IR camera frame rate of 30 frames per second.

Unlike traditional methods, FITA facilitates in situ measurement of irradiated samples within a vacuum, eliminating the necessity to disrupt the experimental setup. This approach is particularly advantageous for accelerator-based irradiation experiments, where the only additional components required are an infrared (IR) camera and an IR transparent viewport, thus enabling thermal property measurements with minimal interruption to the ongoing experimentation. FITA employs a focused proton beam to generate a localized hot spot on the sample surface. The resulting temperature distribution and heat dissipation are captured via the IR camera, this in combination with FEA allows for measuring thermal properties.

FITA offers several advantages: (a) Remote and non-destructive testing: This method is ideal for university researchers handling radioactive materials, as it eliminates the need for direct contact. (b) Integrated testing: The approach can be combined with ion irradiation. A single specimen can yield data on thermal property changes as a function of radiation damage level, simply by adding repeated irradiation and analysis. (c) Minimal thickness requirements: Unlike traditional methods, this technique does not require strict thickness uniformity, although surface smoothness must be maintained at an acceptable level.

Our previous study demonstrated the feasibility of using this technique on quartz. However, quartz is not a good thermal conductor. The applicability of the technique to a wider range of materials remained unclear. Furthermore, the constraints and limitations of the technique, as well as the factors that can mitigate these constraints, need to be thoroughly discussed. This is the purpose of the present study. As will be discussed later, the performance of FITA is influenced by the resolution and frame rate of the IR camera, which imposes constraints on the types of materials that can be effectively analyzed, especially those with relatively high thermal conductivities. To address these practical limitations, the present study explores the boundaries of FITA’s applicability through a combination of experimental investigations and finite element analysis model using ANSYS. By conducting a comprehensive series of simulations across a wide range of thermal property parameters, this work aims to systematically evaluate how the resolution and frame rate of IR cameras impact FITA’s effectiveness in analyzing different materials. The insights gained from this study will inform future developments and expand the potential applications of FITA in advanced nuclear materials research.

## 2. Experimental Procedure

**Figure 1:**
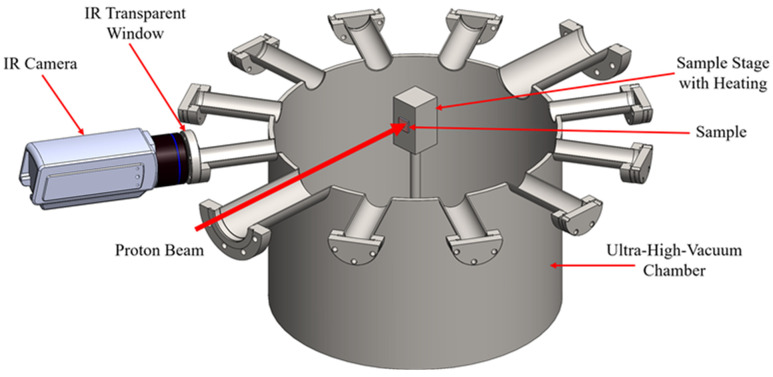
Schematic of the FITA setup, featuring an ultra-high-vacuum chamber, a heated sample stage, a proton beam for localized heating, and an IR camera monitoring thermal response through an IR-transparent window.

Figure 1 schematically illustrates the irradiation chamber configuration. A 1.7 MV tandem accelerator at Texas A&M University is used to produce a proton beam of 2 MeV. The proton beam, with a diameter of 0.8 mm, bombards the specimen, which is larger than the beam spot size. The hot stage, composed of molybdenum, measures 20 × 54 × 18 mm^3^. The specimen is mounted on the hot stage, allowing experiments to be conducted at elevated temperatures of up to 1200 °C. The maximum achievable temperature is limited by the heater’s maximum power and the chamber’s heat dissipation capacity. Special precautions must be taken when the temperature exceeded 900 °C to ensure proper vacuum sealing of the target chamber. To demonstrate the validity of the FEA simulations in this report one measurement on UO_2_ fuel pellets at 200 °C will be conducted.

Temperature mapping during the experiments was achieved using an FLIR T865-24 infrared camera (Teledyne FLIR LLC, Wilsonville, OR, USA). The infrared (IR) imaging system operates at a frame rate of 30 frames per second. The IR signals pass through an IR transparent window before reaching the camera. This window is made of ZnSe with 60–70% transmission in the 7.5 to 14.0 μm range was used, matching the camera’s spectral sensitivity [7]. All the other ports in the chamber are covered to minimize light inside the chamber. Due to the partial absorption by the ZnSe window, the temperature readings from the IR camera require calibration. This is achieved by comparing the temperature readings from the IR camera with those obtained from thermocouples attached to the specimen. The calibration not only compensates for signal loss caused by the IR window but also accounts for the emissivity of the sample. The emissivity depends on the material and surface roughness of the sample. Therefore, each sample must be calibrated individually to ensure accurate temperature measurements.

## 3. Modeling Procedure

Thermal properties can be determined under two conditions: when the ion beam is active (on) and inactive (off). The analysis of temperature evolution in each scenario is governed by different equations. When the beam is active, the temperature evolution is described by the following:

$$\frac{\partial T}{\partial t}=\alpha \nabla^{2}T+\frac{q_{beam}}{\rho C_{p}}\;$$

Conversely, when the beam is turned off, the equation simplifies to

$$\frac{\partial T}{\partial t}=\alpha \nabla^{2}T$$

Here, T is temperature, t is time, α is the thermal diffusivity, $\rho C_{p}\;$ is the volumetric heat capacity at constant pressure, and $q_{beam}$ is the volumetric heat gain from the beam. At the boundary, there is a radiative heat flux leaving the surface due to blackbody radiation, as described by the following:

$$-k\frac{\partial T}{\partial n}=\sigma \epsilon \left(T_{s}^{4}-T_{\infty }^{4}\right)$$

where k is the thermal conductivity, σ is the Stefan-Boltzmann constant, ϵ is the emissivity, $T_{s}$ is the surface temperature, and $T_{\infty }$ is the ambient temperature (assumed to be zero, since the sample surface faces vacuum). In the present study, σ takes the value of 5.670374419 × 10^−8^ W⋅m^−2^⋅K^−4^. ϵ is approximated as 1.

The 2 MeV proton beam used as a heating source has limited penetration depth. In metals, the proton’s projected range is typically on the order of several tens of microns. The power deposition along the proton penetration depth was calculated using the Monte Carlo simulation code SRIM (Stopping and Range of Ions in Matter) by extracting electronic stopping power and nuclear stopping power curves and applying the following equation:

$$q_{beam}=\frac{I}{Ae}\frac{dE}{dx}$$

where I is the beam current, A is the cross-sectional area of the beam, e is the electron charge, and $\frac{dE}{dx}$ is the stopping power of the proton beam.

Obtaining stopping power as a function of depth is not straightforward and requires several steps, as it is not directly available from SRIM [8]. The procedure is as follows: (1) Obtaining projected range: SRIM provides the projected range of protons at different initial bombardment energies. (2) Obtaining stopping power: Both electronic and nuclear stopping powers at different energies are obtained. (3) Depth-to-energy conversion: For a given incident proton energy (e.g., 2 MeV), the projected range is determined. Using the range table, the energy of a proton at a specific depth can be estimated. For example, a 2 MeV proton in Fe has a projected range of about 20 microns. At a depth of 5 microns, the proton must penetrate an additional 15 microns to reach its final resting depth. This penetration distance can be converted to a specific energy based on the range table/curve obtained from the first step. By repeating this for all depth points, a local energy value can be approximated for each depth. (4) Obtaining stopping power deposition curve: The local energy values are converted to stopping powers using the table/curve obtained in the second step.

The heating source distribution obtained from these steps is then used in the finite element simulation. For the study, the FEA was performed using the commercial software Ansys 2023-R2. Additionally, the amount of power released from conduction and blackbody radiation can be easily incorporated into the FEA model. Heat loss by convection was not included in the model since the experiments took place in a high-vacuum environment.

## 4. Results

**Figure 2:**
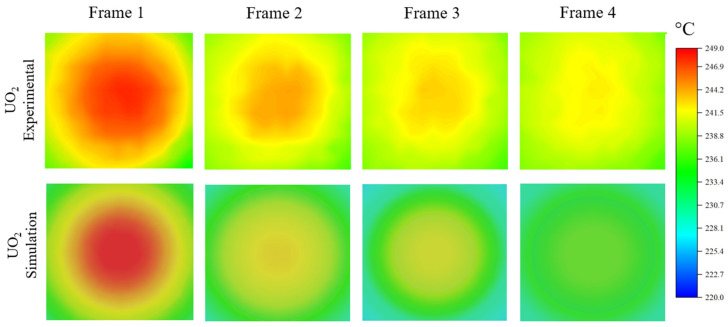
(**Top**) Experimentally obtained temperature evolution of UO_2_ at *t* = 0, 1/300, 2/300, and 4/300 s after the beam is turned off. (**Bottom**) Modeling-predicted temperature evolution using thermal diffusivity values obtained from the literature [9].

Figure 2 presents the temperature evolution of UO_2_ fuel pellets on a stage heated to 200 °C, capturing both experimental (top) and model-predicted (bottom) distributions. The starting time, *t* = 0, corresponds to the moment when beam-induced heating has already reached a steady state, and the beam is about to be turned off. This steady state is typically achieved after the beam has been on the sample for a few seconds. The hot spot diameter is approximately 1.5 mm. At the center of the beam spot, the temperature reaches approximately 250 °C, while the surrounding area outside the beam spot is at approximately 220 °C. At *t* = 0, the beam is turned off. The second frame corresponds to *t* = 1/30 s after the beam is turned off. At this point, the hot spot becomes weaker and begins to shrink in size. By 4/30 s, the hot spot has almost completely faded.

The bottom portion of Figure 2 shows corresponding FEA simulation results, using theoretical thermal diffusivity values obtained from the literature [9]. The FEA model replicates the entire heated stage assembly, including the sample, with precise adherence to the experimental dimensions. The cylindrical UO_2_ sample features a diameter of 5 mm and a thickness of 2 mm. The 0.8 mm diameter proton beam, which influences a 25-μm thick region, is simulated as an internal heat source. The temperature evolution of the hot spot from the simulation is in good agreement with the experimental observations, validating the modeling setup within a margin of 2 °C.

The resolution of these measurements and the ability to characterize specific materials largely depend on a combination of factors including the material’s density, thermal conductivity, and specific heat. Particularly, materials with high thermal conductivity pose a significant challenge for this method, as heat dissipation occurs very rapidly. This rapid dissipation can prevent the infrared (IR) camera from capturing the transient temperature changes accurately within its limited frame rate.

**Figure 3:**
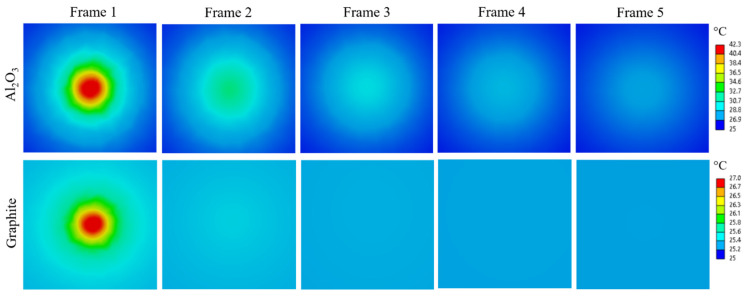
(**Top**) Modeled temperature evolution of the hot spot induced by 2 MeV proton bombardment in aluminum oxide. (**Bottom**) Modeled temperature evolution under the same conditions for graphite.

Figure 3 compares the simulated temperature evolution for aluminum oxide, representing a good example for a low thermal conductivity material, and pure graphite representing a relatively high thermal conductivity material. The thermal properties used in the simulations for both materials were sourced from the Ansys Granta Materials database [10]. A beam spot diameter of 1 mm and a beam power density of 20 W/mm^3^ was used. The first frame for both materials corresponds to the moment when the beam is turned off. Aluminum oxide, with a thermal conductivity of 13.75 W/mK, shows a sharp gradient and slow heat dissipation, keeping the hot spot visible up to *t* = 5/30 s. Graphite, with a higher conductivity of 138.6 W/mK, dissipates heat rapidly, with the hot spot becoming nearly indistinguishable by *t* = 2/30 s. This comparison underscores the influence of thermal diffusivity and thermal conductivity on the temperature evolution and the method’s ability to capture these changes.

To identify the range of thermal properties measurable using a specific frame rate, a broad parameter space below volumetric heat capacity ($\rho C_{p}$) of 4 × 10^7^ J/m^3^K was screened. The thermal conductivity values between 0.01 and 500 W/mK and thermal diffusivity values between 1 × 10^−8^ and 5 × 10^−4^ m^2^/s were considered. This range covers most known materials with the exception of ultra-high thermal conductivity materials. Utilizing a screening algorithm, 750 points within this parameter space were generated and subsequently modeled using a transient thermal simulation in Ansys. A simplified model with a sample dimensions of 20 mm^3^ was considered, with a beam spot diameter of 1 mm and a beam power density of 20 W/mm^3^. The beam-on time was maintained at 10 s to capture the temperature evolution from the beam spot.

**Figure 4:**
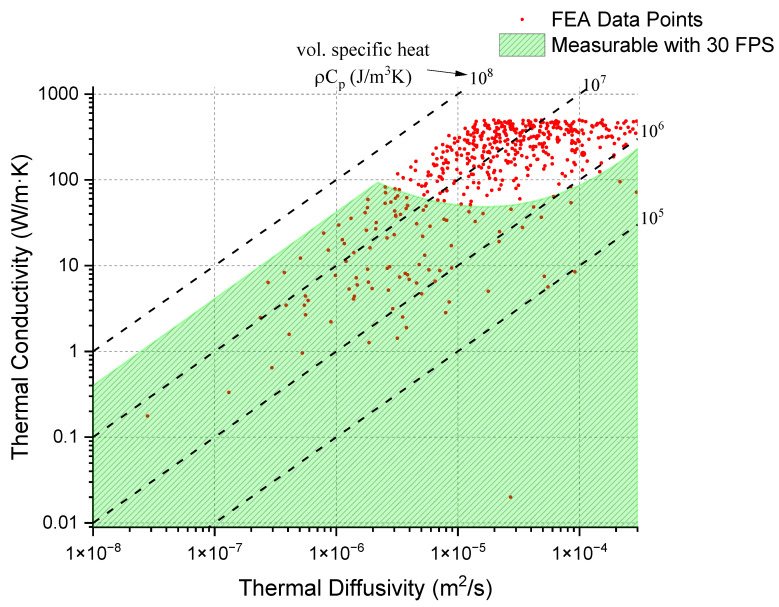
Map of thermal conductivity and thermal diffusivity for materials randomly selected for simulation. The green-colored region represents the range where the material’s thermal properties are measurable, based on the criterion that a hot spot with a temperature difference of >0.5 °C remains after 5/30 s following the beam being turned off.

The collected data helped define the practical limitations for experimental measurements limited by the frame rate of the FLIR T865-24 IR camera. This camera operates at a fixed frame rate of 30 frames per second (FPS) with a temperature resolution of 0.5 °C. In Figure 4, the green-colored region delineates conditions under which it is feasible to measure thermal properties using this setup. Feasibility is specifically defined as the camera’s ability to detect a hot spot where the temperature is at least 0.5 °C higher than its surrounding area at 5/30 s after the beam is turned off. This criterion ensures that sufficient data points are collected before the hot spot dissipates, allowing for an accurate calculation of the material’s thermal properties.

The behavior of a hot spot’s temperature is largely influenced by the thermal conductivity and specific heat capacity of the material. Materials with higher thermal conductivity dissipate heat more efficiently, leading to a lower temperature rise at the hot spot. When thermal diffusivity is not very high, the temperature increase at the hot spot is particularly sensitive to the specific heat capacity. However, when thermal diffusivity is rather large, heat spreads rapidly across the material, which tends to lower the temperature at the hot spot more quickly. In such scenarios, to achieve a higher temperature rise, it becomes necessary to reduce the specific heat capacity, thus compensating for the quick heat dissipation. This correlation is what creates the curved feature observed at the top of the regions in our analysis (the top boundary of the green-colored region in Figure 4). The dashed line illustrated in Figure 4 represents the ratio of thermal conductivity to thermal diffusivity, which directly correlates to specific heat capacity.

**Figure 5:**
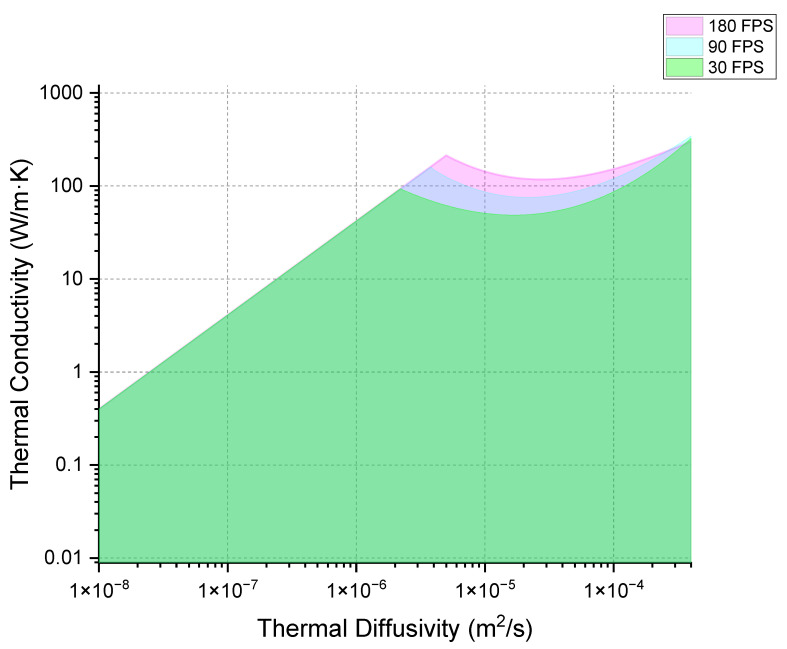
The effect of increasing camera frame rate on the map of measurable thermal conductivity and thermal diffusivity.

If we set the criterion that a temperature rise of >0.5 °C must still exist after five frames, increasing the camera frame rate can expand the range of measurable materials. A faster camera allows for the measurement of materials with quicker heat dissipation if the initial temperature rise of the hot spot remains the same, or materials with the same heat spreading characteristics but a lower initial temperature rise in the hot spot. As a result, the upper limit of detectable thermal conductivity increases. Figure 5 demonstrates how the boundaries for infrared (IR) cameras at 30, 90, and 180 frames per second expand, allowing for the detection of materials with progressively higher thermal conductivity values. It suggests that increasing camera frame rate enhances the characterization capability for materials with high thermal conductivity. However, this gain diminishes in materials with extremely high thermal diffusivity. For various steels and metals commonly used in industry, their thermal diffusivity typically ranges from 1 × 10^−5^ to 1 × 10^−4^ m^2^/s, which falls within the region where increasing camera frame rate can still improve resolution.

## 5. Discussion

**Figure 6:**
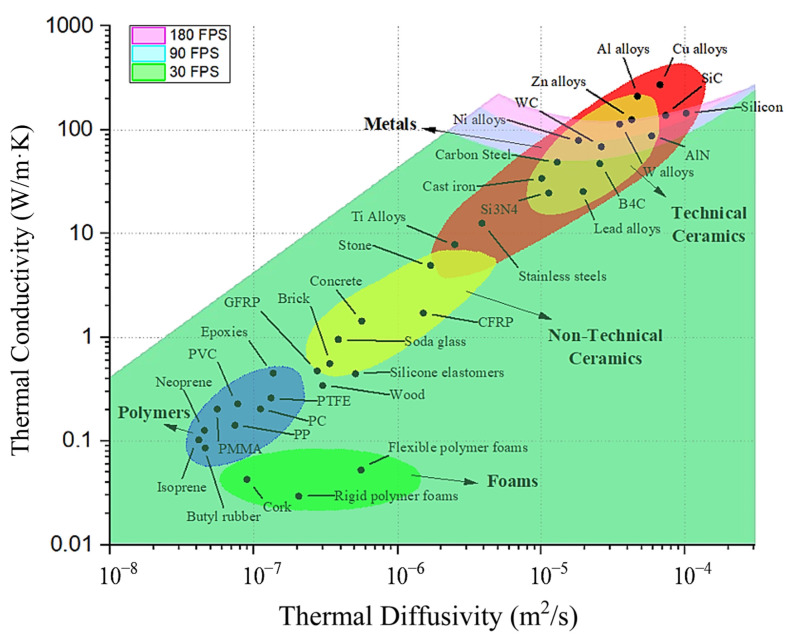
The map of measurable thermal conductivity and thermal diffusivity, overlaid with the properties of materials commonly used in industrial applications. The properties were replotted with permission from Reference [11].

Figure 6 overlays the map of measurable thermal properties with material properties collected from the literature [11]. This demonstrates the extent to which the proposed technique can be applied to materials of industrial interest. This map captures a significant range of materials, including polymers with thermal conductivities below 1 W/mK and composite materials below 10 W/mK, along with most metals and nearly all ceramics. Materials that fall beyond the measurable map include high-conductivity materials like certain copper alloys and aluminum alloys.

**Figure 7:**
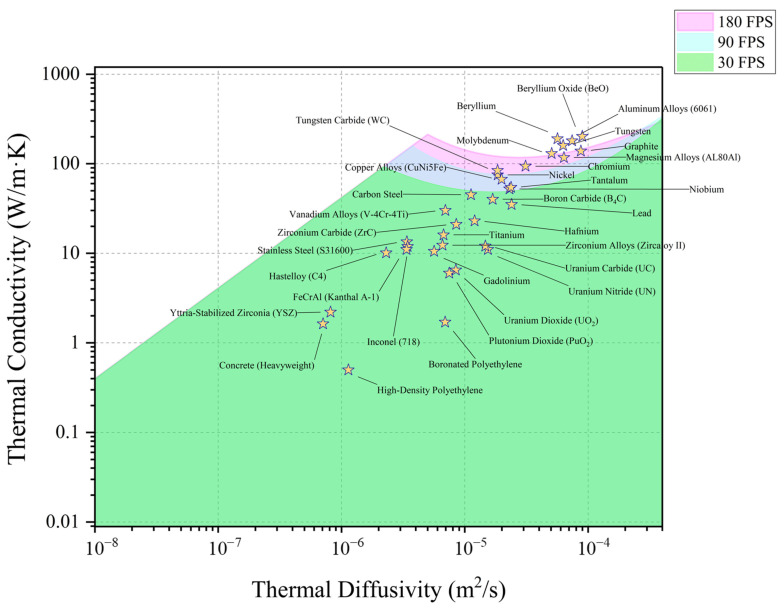
The map of measurable thermal conductivity and thermal diffusivity, overlaid with the properties of nuclear materials commonly used in nuclear industry [10,11,12,13,14,15,16,17,18,19,20,21,22,23,24,25].

Further extending our analysis to the nuclear industry, Figure 7 shows the measurable map overlaid with materials frequently employed in this sector [10,11,12,13,14,15,16,17,18,19,20,21,22,23,24,25]. It covers traditional nuclear fuels like UO_2_ and advanced high-temperature fuels such as UC and UN, as well as fuel cladding materials like Zircaloy and structural materials like S31600 steels used in-core, and out-of-core materials like Inconel. Materials that are challenging to characterize include tungsten, aluminum alloys, and beryllium oxide. These materials lie slightly above the measurable zone but could potentially be covered if the IR camera frame rate is further increased. Tungsten is of particular interest as a candidate material for the first wall in fusion reactors.

Changes in thermal properties can occur as a result of radiation-induced microstructural alteration and transmutation. While transmutation does not occur during ion bombardment, significant microstructural and microchemical changes can occur. Significant degradation can also occur when microstructural defects such as microcracking start developing. Microcracks are particularly prone to forming along grain boundaries when irradiation damage is combined with gas atom injection, as observed in tungsten when it is used as the first wall material in fusion reactors [26]. Radiation damage can significantly alter thermal properties of ceramics, where phonons (or electrons at high temperatures) play a dominant role in heat conduction [26]. Phonon scattering caused by radiation-induced defects is particularly effective in degrading the thermal properties of ceramics.

It is well known that voids and bubbles induced by displacive irradiation of austenitic stainless steels lead to significant changes in many physical properties, including lattice parameter, bulk density, elastic moduli, Poisson’s ratio, electrical/thermal conductivities and ultrasonic velocity with measurable consequences on dimensional and mechanical stability [27]. Thermal resistivity increases ~1% for each percent of swelling. Changes in resistivity and other properties can also occur when elements such as C and Si come out of solution as a result of radiation to form precipitates such as carbides [27]. Such radiation-induced changes in microstructure and microchemistry can be viewed as complications to the use of the FITA, but on the other hand, FITA may be used as a tool to detect the onset and growth of these radiation-induced components.

The proposed method is particularly valuable for studying the thermal property degradation of nuclear materials under harsh reactor environments. Since the technique utilizes an ion irradiation beam as the analysis tool, it facilitates seamless incorporation of irradiation into the characterization process. By switching the focused analysis beam to a rastering irradiation beam, radiation damage can be introduced over a larger area. This flexibility makes it highly feasible for accelerator systems to alternate between thermal property measurements and radiation damage introduction. Consequently, the thermal property changes as a function of damage level can be studied using a single specimen.

Additionally, the technique allows for thermal property characterization along different directions. For materials with specific crystal orientations or textures, thermal properties along various directions may result in temperature hot spots that exhibit directional dependency rather than isotropic circular spreading patterns. For single-crystal materials, the specific crystal orientation of samples can be easily determined using channeling Rutherford Backscattering Spectrometry (RBS), provided that the target stage is mounted on a multi-axial goniometer.

The method can also be used to study possible phase changes under irradiation. If such phase changes occur beyond a certain damage level or elevated temperature, thermal property measurements can act as a phase-monitoring tool. Furthermore, the proposed technique can be extended into a mapping method to visualize thermal properties or phase distributions. This can be achieved by using a mask with a matrix of apertures. For example, a 10 × 10 aperture matrix over a mask can divide a large irradiation beam spot into multiple smaller beam spots. The resulting IR mapping enables simultaneous measurement of thermal properties as a function of location.

The accuracy and repeatability of FITA are quite high and are expected to be better than the traditional laser flash analysis (LFA) technique, considering the following factors: The accuracy of LFA is sensitive to surface roughness, sample thickness errors, the uniformity of laser heating, and contact resistance. None of these factors affect the FITA technique since it is a non-contact measurement. Even if beam heating introduces non-uniformity in FITA, thermal properties can still be extracted using actual temperature mapping, with non-uniformity accounted for in the analysis.

There is no concern regarding repeatability. Repeated ion irradiation over the same region may introduce irrecoverable radiation damage and changes in thermal properties. However, thermal property analysis is not necessarily limited to the beam spot region. The method can be applied to any region as long as a temperature gradient can be established, regardless of whether it is within the beam spot size or outside the beam spot region. In fact, it is feasible to obtain a thermal property mapping of both irradiated and unirradiated regions, including variations observed under repeated measurements. Considering this, thermal property mapping based on the analysis of the entire temperature pattern evolution provides more comprehensive information than a single-spot analysis.

There is no threshold on the maximum number of irradiations allowed, as thermal property data can be extracted from regions that are not directly bombarded. However, care must be taken if beam contamination occurs during prolonged experiments. Surface contamination may influence emissivity and affect the accuracy of temperature mapping. To address this concern, maintaining a high vacuum is always preferred, and techniques such as liquid nitrogen trapping for vacuum improvement should always be considered.

The technique could be applied to heterogeneous materials, such as layered materials with different thermal properties, provided that the spatial size and property differences are within an appropriate range. This can be achieved by either tilting the samples or adjusting the proton beam energy to introduce heating sources at different locations. Finite element analysis may enable the extraction of depth-dependent thermal properties by fitting all spectra.

Regarding the IR window, the current study uses ZnSe, which exhibits a relatively good transmission efficiency (~70%) over a wide wavelength range [28]. ZnSe can be used for wavelengths up to approximately 20 μm. Other window materials, such as BaF_2_, have higher transmission (>90%), but their maximum operable wavelength is around 13 μm [28]. CaF_2_ can be used up to about 8 μm, but its transmission is not constant [28]. Sapphire has a high transmission, close to 90%, but its operable wavelength is only about 6 μm, limiting its application mainly to high-temperature environments [28]. Depending on the temperature range, material selection needs to consider transmission performance across the entire temperature range. Overall, ZnSe is an excellent choice, given its broad applicable temperature range. Transmission varies for different materials, and these effects have been accounted for in the calibration process.

As a new technique for measuring thermal properties, FTIA needs to be fully evaluated over a wide temperature range and for various materials. A systematic comparison is currently underway. It is worth pointing out that as a remote, non-contact characterization tool, FTIA obviously exhibits its uniqueness in handling some traditionally difficult materials, such as ice.

## 6. Conclusions

The present study discusses the applicable range of thermal properties for materials that can be characterized using ion beams as a heating method for remote thermal property characterization. Through detailed FEA, we assessed the limitations imposed by factors such as the IR camera’s frame rate and temperature resolution. Our findings show that the current method can characterize a wide range of materials, including the majority of nuclear materials typically used in the nuclear industry. Furthermore, increasing the frame rate of the camera could allow for the capture of rapid thermal transients in materials with higher thermal conductivities, which are currently challenging to measure.

## Data Availability

The data presented in this study are available upon request from the corresponding author due to the large size of the data matrix.
